# Interactions between Non-Physician Clinicians and Industry: A Systematic Review

**DOI:** 10.1371/journal.pmed.1001561

**Published:** 2013-11-26

**Authors:** Quinn Grundy, Lisa Bero, Ruth Malone

**Affiliations:** 1 Department of Social and Behavioral Sciences, School of Nursing, University of California, San Francisco, San Francisco, California, United States of America; 2 Department of Clinical Pharmacy, School of Pharmacy, University of California, San Francisco, San Francisco, California, United States of America; 3 Institute for Health Policy Studies, School of Medicine, University of California, San Francisco, San Francisco, California, United States of America; Harvard University, Brigham and Women's Hospital, United States of America

## Abstract

**Background:**

With increasing restrictions placed on physician–industry interactions, industry marketing may target other health professionals. Recent health policy developments confer even greater importance on the decision making of non-physician clinicians. The purpose of this systematic review is to examine the types and implications of non-physician clinician–industry interactions in clinical practice.

**Methods and Findings:**

We searched MEDLINE and Web of Science from January 1, 1946, through June 24, 2013, according to PRISMA guidelines. Non-physician clinicians eligible for inclusion were: Registered Nurses, nurse prescribers, Physician Assistants, pharmacists, dieticians, and physical or occupational therapists; trainee samples were excluded. Fifteen studies met inclusion criteria. Data were synthesized qualitatively into eight outcome domains: nature and frequency of industry interactions; attitudes toward industry; perceived ethical acceptability of interactions; perceived marketing influence; perceived reliability of industry information; preparation for industry interactions; reactions to industry relations policy; and management of industry interactions. Non-physician clinicians reported interacting with the pharmaceutical and infant formula industries. Clinicians across disciplines met with pharmaceutical representatives regularly and relied on them for practice information. Clinicians frequently received industry “information,” attended sponsored “education,” and acted as distributors for similar materials targeted at patients. Clinicians generally regarded this as an ethical use of industry resources, and felt they could detect “promotion” while benefiting from industry “information.” Free samples were among the most approved and common ways that clinicians interacted with industry. Included studies were observational and of varying methodological rigor; thus, these findings may not be generalizable. This review is, however, the first to our knowledge to provide a descriptive analysis of this literature.

**Conclusions:**

Non-physician clinicians' generally positive attitudes toward industry interactions, despite their recognition of issues related to bias, suggest that industry interactions are normalized in clinical practice across non-physician disciplines. Industry relations policy should address all disciplines and be implemented consistently in order to mitigate conflicts of interest and address such interactions' potential to affect patient care.

*Please see later in the article for the Editors' Summary*

## Abstract 2

In a systematic review of studies of interactions between non-physician clinicians and industry, Quinn Grundy and colleagues found that many of the issues identified for physicians' industry interactions exist for non-physician clinicians.

*Please see later in the article for the Editors' Summary*

## Editors' Summary

**Background:**

Making and selling health care goods (including drugs and devices) and services is big business. To maximize the profits they make for their shareholders, companies involved in health care build relationships with physicians by providing information on new drugs, organizing educational meetings, providing samples of their products, giving gifts, and holding sponsored events. These relationships help to keep physicians informed about new developments in health care but also create the potential for causing harm to patients and health care systems. These relationships may, for example, result in increased prescription rates of new, heavily marketed medications, which are often more expensive than their generic counterparts (similar unbranded drugs) and that are more likely to be recalled for safety reasons than long-established drugs. They may also affect the provision of health care services. Industry is providing an increasingly large proportion of routine health care services in many countries, so relationships built up with physicians have the potential to influence the commissioning of the services that are central to the treatment and well-being of patients.

**Why Was This Study Done?:**

As a result of concerns about the tension between industry's need to make profits and the ethics underlying professional practice, restrictions are increasingly being placed on physician–industry interactions. In the US, for example, the Physician Payments Sunshine Act now requires US manufacturers of drugs, devices, and medical supplies that participate in federal health care programs to disclose all payments and gifts made to physicians and teaching hospitals. However, other health professionals, including those with authority to prescribe drugs such as pharmacists, Physician Assistants, and nurse practitioners are not covered by this legislation or by similar legislation in other settings, even though the restructuring of health care to prioritize primary care and multidisciplinary care models means that “non-physician clinicians” are becoming more numerous and more involved in decision-making and medication management. In this systematic review (a study that uses predefined criteria to identify all the research on a given topic), the researchers examine the nature and implications of the interactions between non-physician clinicians and industry.

**What Did the Researchers Do and Find?:**

The researchers identified 15 published studies that examined interactions between non-physician clinicians (Registered Nurses, nurse prescribers, midwives, pharmacists, Physician Assistants, and dieticians) and industry (corporations that produce health care goods and services). They extracted the data from 16 publications (representing 15 different studies) and synthesized them qualitatively (combined the data and reached word-based, rather than numerical, conclusions) into eight outcome domains, including the nature and frequency of interactions, non-physician clinicians' attitudes toward industry, and the perceived ethical acceptability of interactions. In the research the authors identified, non-physician clinicians reported frequent interactions with the pharmaceutical and infant formula industries. Most non-physician clinicians met industry representatives regularly, received gifts and samples, and attended educational events or received educational materials (some of which they distributed to patients). In these studies, non-physician clinicians generally regarded these interactions positively and felt they were an ethical and appropriate use of industry resources. Only a minority of non-physician clinicians felt that marketing influenced their own practice, although a larger percentage felt that their colleagues would be influenced. A sizeable proportion of non-physician clinicians questioned the reliability of industry information, but most were confident that they could detect biased information and therefore rated this information as reliable, valuable, or useful.

**What Do These Findings Mean?:**

These and other findings suggest that non-physician clinicians generally have positive attitudes toward industry interactions but recognize issues related to bias and conflict of interest. Because these findings are based on a small number of studies, most of which were undertaken in the US, they may not be generalizable to other countries. Moreover, they provide no quantitative assessment of the interaction between non-physician clinicians and industry and no information about whether industry interactions affect patient care outcomes. Nevertheless, these findings suggest that industry interactions are normalized (seen as standard) in clinical practice across non-physician disciplines. This normalization creates the potential for serious risks to patients and health care systems. The researchers suggest that it may be unrealistic to expect that non-physician clinicians can be taught individually how to interact with industry ethically or how to detect and avert bias, particularly given the ubiquitous nature of marketing and promotional materials. Instead, they suggest, the environment in which non-physician clinicians practice should be structured to mitigate the potentially harmful effects of interactions with industry.

**Additional Information:**

Please access these websites via the online version of this summary at http://dx.doi.org/10.1371/journal.pmed.1001561.

- This study is further discussed in a *PLOS Medicine* Perspective by James S. Yeh and Aaron S. Kesselheim
- The American Medical Association provides guidance for physicians on interactions with pharmaceutical industry representatives, information about the Physician Payments Sunshine Act, and a toolkit for preparing Physician Payments Sunshine Act reports
- The International Council of Nurses provides some guidance on industry interactions in its position statement on nurse-industry relations
- The UK General Medical Council provides guidance on financial and commercial arrangements and conflicts of interest as part of its good medical practice website, which describes what is required of all registered doctors in the UK
- Understanding and Responding to Pharmaceutical Promotion: A Practical Guide is a manual prepared by Health Action International and the World Health Organization that schools of medicine and pharmacy can use to train students how to recognize and respond to pharmaceutical promotion.
- The Institute of Medicine's Report on Conflict of Interest in Medical Research, Education, and Practice recommends steps to identify, limit, and manage conflicts of interest
- The University of California, San Francisco, Office of Continuing Medical Education offers a course called Marketing of Medicines

## Introduction

Although health professionals interface with industry daily and often operate within health systems owned by corporations, a tension exists between the legally mandated corporate mission to maximize profits for shareholders and the ethics underlying professional practice [1]. Relationships between clinicians and the pharmaceutical industry have come under scrutiny because of their potential for conflict of interest and resultant impacts on the cost, quality, and safety of health care [2]. These relationships may result in increased prescription rates, particularly of new, heavily marketed medications, which are often more expensive than their generic counterparts and are more likely to be recalled for safety reasons [2],[3]. Concerns have also been raised regarding the “corporatization” of health care, the appropriation of increasing proportions of the health sector by corporate industry, and the accompanying routinization, standardization, and fragmentation of health care services, processes that are fundamentally changing the way that health professionals practice [4]–[6].

Most research on these topics has focused on physicians' relationships with the pharmaceutical industry. However, multiple industries' marketing efforts, including those of the medical device, information technology, and infant formula industries, may target health professionals across disciplines and practice settings [7]–[10]. This review defines “industry” as the major corporations that produce health care goods and services as well as their public relations firms and associated scientific entities. Though often examined individually, similarities in corporate tactics such as the suppression or misrepresentation of scientific evidence, political lobbying techniques, sophisticated marketing strategies, and the use of front groups have been noted across different industries, suggesting that similar patterns of interactions with health professionals may exist [11]–[15].

The Physician Payments Sunshine Act was passed as part of the United States' Affordable Care Act in an effort to bring greater transparency to physicians' financial relationships with industry [16]. This legislation requires US manufacturers of drugs, devices, and medical supplies covered under public insurance programs such as Medicare to disclose all payments and gifts made to physicians and teaching hospitals [16]. However, all other health professionals, including those with prescriptive authority such as Doctors of Pharmacy (PharmDs), Physician Assistants (PAs), and Nurse Practitioners (NPs), are omitted from this legislation.

In this context, nurses, pharmacists, PAs, and other allied health professionals may be viewed as powerful and desirable partners for industry, making them “soft targets” for marketing [7] and vulnerable to the same conflicts of interest that raised concerns about physicians [17]. Because of multiple health policy developments that prioritize primary care, multidisciplinary care models, and increased access to care, mid-level prescribers such as PAs, NPs, and other advanced practice nurses are projected to grow in number and to fulfill growing demands for primary care services [18],[19]. The restructuring of health care to emphasize integrated multidisciplinary teams has prompted calls to maximize the contributions of often-underutilized professionals such as pharmacists to promote medication management [20]. Registered Nurses (RNs), though without prescriptive authority, also exert influence over treatment decision-making in a variety of ways [21]. RNs may be involved in purchasing decisions, including selecting products for nursing care, choosing treatments from among standing orders, and evaluating products on behalf of an institution, and may facilitate industry representatives' access to prescribers and patients. Thus, several types of non-physician clinicians are involved in the same types of decision-making that raised concerns leading to the new disclosure policies for physicians. Despite their involvement, we were unable to locate any previous reviews examining such non-physician clinician–industry interactions.

The purpose of this systematic review was to examine the nature and implications of non-physician clinician–industry interactions in clinical practice. The research questions addressed were: (1) What types of interactions between non-physician clinicians and industry in clinical practice have been described? (2) What is known about these interactions? In this review, the term clinician is used inclusively to refer to all disciplines in the included studies. The participants (non-physician clinicians) and “interventions” (industry interactions) were defined a priori; we included all outcomes reported in the identified research articles.

## Methods

### Study Selection Criteria

We searched for all original research articles published since January 1, 1946, that addressed non-physician clinician–industry interactions in clinical practice (our search followed PRISMA guidelines; see Text S1). Nonempirical articles, including theoretical essays, literature reviews, editorials, opinion pieces, and letters to the editor, were excluded. Clinicians eligible for inclusion were: RNs; advanced practice nurses with prescriptive authority (here referred to as nurse prescribers) such as NPs, Clinical Nurse Specialists, midwives, and Certified Registered Nurse Anesthetists; PAs; pharmacists; dieticians; and physical or occupational therapists; trainee samples were excluded. Studies were included if they reported clinicians' exposure to industry; industry exposure was defined as meetings with sales representatives; receipt of gifts, payments, or promotional materials including samples; or attendance at industry-sponsored education. There were no limits placed on language of report, country, study design, or outcomes measured. Articles were excluded if they comprised physician-only samples; solely addressed industry interactions in the context of research, including clinical trials; examined direct-to-consumer advertising; examined industry relations with organizations; were theoretical; or performed model testing (without hypothesis testing).

### Data Sources and Searches

MEDLINE and Web of Science databases were searched for articles published from January 1, 1946, through June 24, 2013. Three domains of key words were joined by the AND operator: (1) the ethical phenomenon; (2) types of health professionals, and (3) key words for industry. Medical Subject Headings (MeSH) terms and free text were both utilized. The principle search strategy, developed in consultation with a medical librarian, was as follows:

(“conflict* of interest” OR ethics[MeSH] OR marketing[MeSH]) AND (nurse[MeSH] OR nursing OR nurses OR physician[MeSH] OR doctor OR physicians OR pharmacist OR pharmacists OR therapist) AND (corporate OR corporation OR corporations OR manufacturer OR industry[MeSH] OR company OR companies)

Once relevant articles were identified, their reference lists were searched for additional articles.

### Study Selection

Q. G. conducted the search, screened for relevant titles and abstracts, and reviewed reference lists for additional titles. Both Q. G. and R. M. assessed the full texts of 43 records for inclusion in the study, with L. B. reviewing discrepancies, and final selection was made by consensus. L. B. advised on all phases of the review. Sixteen articles representing 15 studies were determined to meet the inclusion criteria.

### Data Collection and Synthesis

Data were collected on the following study characteristics upon which frequencies were calculated: country of origin; year of publication; study design; population sampled; industry examined; sampling method; sampling frame; and presence and type of disclosure statements. Because of the heterogeneity of study design, the absence of experimental studies, the lack of standardized measurement tools for outcome assessment, and the inclusion of qualitative reports, meta-analysis of data from retrieved studies was not appropriate. Using an author-generated data collection form, Q. G. abstracted study data. For accuracy, this process was repeated two times by the same author, Q. G.

Studies were then grouped thematically for descriptive analysis based upon outcomes reported. An inductive approach was taken to study synthesis by grouping outcomes measured or reported into domains; these domains were not identified a priori, but rather all outcomes were included and grouped thematically as they were abstracted. This synthesis was achieved through first identifying all outcomes measured (or described, in the case of qualitative studies) and then identifying commonalities. Outcome domains were revised, added, and synthesized as data were abstracted until all outcomes related to the research question had been captured. All three authors participated in identifying and refining these outcome domains. Findings within each outcome domain were then analyzed descriptively and reported using a narrative approach.

### Methodological Rigor

Since the only studies identified were cross-sectional survey, interview, and focus group designs (with 27% [*n = *4/15] of included studies being qualitative studies), established tools for assessing methodological rigor were not appropriate. While such tools exist, their value for critical appraisal of qualitative research is contested, as the insights generated may not be associated with ratings of methodological quality [22],[23]. Therefore, because of the heterogeneity and cross-sectional, descriptive, and largely exploratory nature of the study designs, the only measures of methodological rigor the authors could consistently appraise were study design, sampling strategy, and sample size. This information is reported alongside individual study results and in Table 1 to aid the reader in assessing the risk of bias in study findings.

**Table 1:** Characteristics of included studies.

Study (Year of Publication) [Reference], Country	Design	*n*	Population; Sampling Strategy	Industry	Outcomes Measured
Pinckney et al. (2011) [39], US	Cross-sectional	206	Multidisciplinary (physicians, NPs, PAs); convenience	Pharmaceutical	Nature and/or frequencyAttitudes toward industryPerceived influence
Ladd et al. (2010) [25], US	Cross-sectional	263	NPs; random	Pharmaceutical	Nature and/or frequencyAttitudes toward industryEthical acceptabilityPerceived influencePerceived reliability
Mahoney and Ladd (2010) [41] US	Focus groups	14	NPs (gerontological); purposive	Pharmaceutical	Attitudes toward industry
Crigger et al. (2009) [34], US	Cross-sectional	84	NPs (family practice); systematic random	Pharmaceutical	Nature and/or frequencyEthical acceptabilityPerceived influencePreparation for interactions
Fischer et al. (2009) [26], US	Focus groups	61	Multidisciplinary (physicians, PAs, NPs, PharmDs); convenience	Pharmaceutical	Nature and/or frequencyAttitudes toward industryPerceived influencePerceived reliabilityPolicy reactionManaging interactions
Jutel and Menkes (2009) [27], New Zealand	Cross-sectional	120	RNs; convenience	Pharmaceutical	Nature and/or frequencyEthical acceptabilityPerceived influencePerceived reliability
Clauson et al. (2008) [35], US	Cross-sectional	92	Nurse prescribers; convenience	Pharmaceutical	Nature and/or frequencyAttitudes toward industryPerceived reliabilityManaging interactions
McInnes et al. (2007) [29], UK	Cross-sectional	669	Multidisciplinary (general practitioners, nurses, dieticians, midwives); convenience	Infant formula	Nature and/or frequencyPerceived reliabilityPolicy reaction
Philipp et al. (2007) [38], US	Cross-sectional	51	Multidisciplinary (RNs, lactation consultants, other staff); convenience	Infant formula	Nature and/or frequency
Farthing-Papineau and Peak (2005) [40], US	Cross-sectional	1,640	Pharmacists; stratified random	Pharmaceutical	Attitudes toward industryPerceived reliabilityPolicy reaction
Nolan et al. (2004) [33], US	Cross-sectional	51	Clinical Nurse Specialists (psychiatric); convenience	Pharmaceutical	Nature and/or frequencyAttitudes toward industryPreparation for interactions
Hall et al. (2009) [31]; Hall et al. (2003) [32], UK	Semi-structured interviews	14	District nurses (prescribers); purposive	Pharmaceutical	Nature and/or frequencyAttitudes toward industryPerceived reliabilityPolicy reaction
Aguayo et al. (2003) [36], Togo and Burkina Faso	Multisite cross-sectional	186	Multidisciplinary (physicians, midwives, nurses); purposive	Infant formula	Nature and/or frequencyPreparation for interactions
Backer et al. (2000) [30], US	Comparative case study/ethnography	53	Multidisciplinary (primary care clinicians); purposive	Pharmaceutical	Nature and/or frequencyManaging interactions
Demeritt (1966) [28], US	Cross-sectional	1,080	Pharmacists; convenience	Pharmaceutical	Nature and/or frequencyAttitudes toward industryPerceived reliabilityPerceived influence

## Results

### Study Characteristics

**Figure 1:**
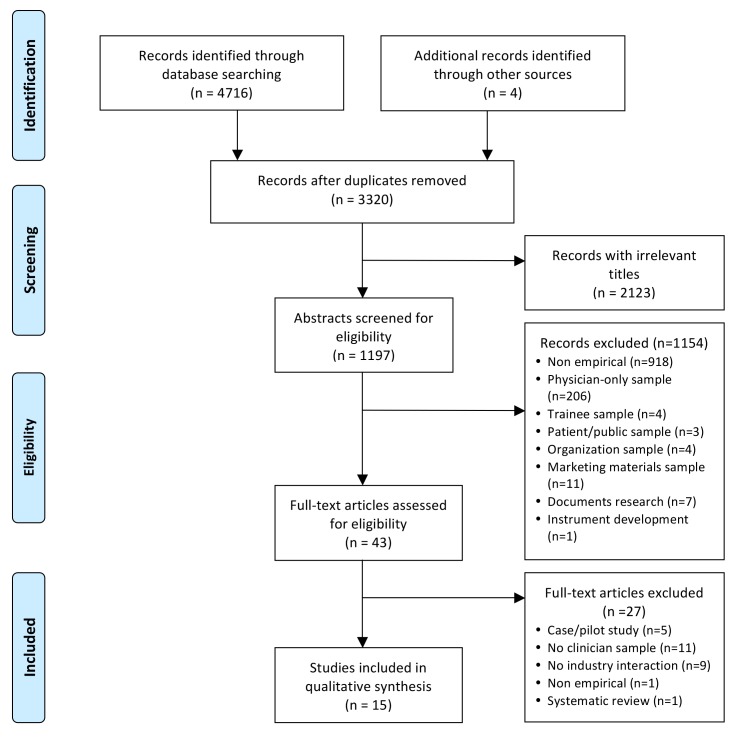
PRISMA flowchart of systematic review search process.

Figure 1 shows the flowchart for article inclusion [24]. The 16 included articles were derived from 15 unique studies. Table 1 shows the characteristics of the included studies. The studies were conducted in four different countries, most in the US, and nearly half were published in the last 5 y (*n = *7/15; 47%). The majority of the studies focused on clinician interactions with the pharmaceutical industry. Seventy-three percent (*n = *11/15) were quantitative cross-sectional surveys; there were no experiments or longitudinal studies. Twenty-seven percent (*n = *4/15) used random sampling methods; 47% (*n = *7/15) used a convenience sample. Participants in most studies were multidisciplinary, including nurse prescribers, RNs, pharmacists, midwives, allied health professionals, and physicians. The sample sizes of the studies ranged from 14 to 1,640.

There were no conflict of interest disclosures for the authors in over half of the included studies (*n = *9/15; 60%). Of those that made declarations, 83% (*n = *5/6 studies) had nothing to declare. Conflict of interest disclosures included: receipt of speakers' fees from pharmaceutical companies and role as expert witness on behalf of the plaintiff in litigation with defendant pharmaceutical companies. Forty percent (*n = *6/15 studies) did not identify their funding source. Of those that did, the majority were funded through public sources; one study was funded by an unrestricted pharmaceutical educational grant.

The studies' findings were categorized into eight domains based upon the outcomes reported (Table 2). The year of study is the publication year because not all studies included the year in which the study was performed.

**Table 2:** Outcome domains.

Outcome Domain	Definition	Number of Studies	Measurement
Nature and/or frequency of industry interactions	Interactions with sales representatives, receipt of gifts, receipt and distribution of product samples, attendance at sponsored education or events	13	Self-report; observation
Attitudes toward industry	Degree to which interactions are judged favorably, or as helpful	9	Self-report
Ethical acceptability of industry interactions	Degree to which interactions are judged to be ethical	3	Self-report
Perceived influence of marketing	Perceptions of the impact of industry interactions on their or their colleagues' practices	6	Self-report; hypothetical scenarios
Perceived reliability of industry information	Perception of bias, objectivity, and comprehensiveness of industry information	8	Self-report
Preparation for industry interactions	Clinicians' formal preparation for how to interact with industry	4	Self-report
Reaction to industry relations policy	Reaction to institutional and professional association policy designed to guide industry–clinician interactions	3	Self-report
Managing industry interactions	Mechanisms used to manage interactions or conflicts of interest	3	Self-report; in-depth qualitative studies

### Nature and Frequency of Interactions with Industry

Thirteen studies assessed the nature and/or frequency of various types of interactions between non-physician clinicians and the pharmaceutical or infant formula industries (Table 2). Interaction type and/or frequency was most commonly measured through self-report; however, a minority of studies (*n = *2) also used direct observation.

#### Representatives

Clinicians met regularly with sales representatives from the pharmaceutical industry; only a minority had eliminated these meetings from their practice. For NPs in 2010, 96% (*n = *252/263) of a nationally representative US sample reported “regular” contact with representatives, though this frequency was undefined [25] (Table 3). On average, US prescribers (Doctors of Medicine [MDs], NPs, PAs, and PharmDs; results were not reported separately by profession) in 2009 met with pharmaceutical representatives nine times a month, with a range of 0–80 [26]. In New Zealand, 14% (*n = *17/120) of a convenience sample of senior RNs received at least biweekly visits from sales representatives in 2009 [27]. Historically, a convenience sample of 1,080 hospital pharmacists in 1966 received an average of 10 visits per week from “detailmen,” with a range from 0 to 43 visits; these visits lasted an average of 15 min [28]. Visits from infant formula sales representatives were experienced infrequently in Glasgow: less than 5% (*n = *32/669) of staff at community health care facilities reported visits from sales representatives in the past 6 mo in 2007 [29]. However, these visits were experienced differentially across disciplines: none of the general practitioners had received visits, but 22% (*n = *11/50) of dieticians and 9% (*n = *20/223) of nurses had [29].

**Table 3:** Reported frequencies of types of industry interactions.

Sample (Location) [Reference]	Type of Interaction					
	Representative Contact	Gifts/Compensation	Meals	Samples	Education (Event)	Education (Materials)
**Nurses**						
NPs (US) [25]	96% reported “regular” contact		49% reported attendance 1–5 times at lunch events, and 64% attendance 1–5 times at dinner events, in the past 6 mo	66%	96% in past 5 y	
NPs (US) [35]		36%				
Family NPs (US) [34]		72%^a^		85%^a^		82%^a^
RNs (New Zealand) [27]	26% reported no contact; 51% responded “monthly or less”; 14% had contact ranging from 2–3 times monthly to 3 or more times per week	75%	55%			100%
**Multidisciplinary**						
MDs, PharmDs, NPs (US) [26]	Mean number of contacts 9 per month					
MDs, PAs, NPs (US) [39]				72%		
General practitioners, nurses, dieticians, midwives, other (UK) [29]	<5% reported having contact in past 6 mo	7%		<0.5%		21%
MDs, nurses, midwives (West Africa) [36]		12%		12% in past 6 mo		16%
RNs, lactation consultants, staff (US) [38]				86%		

a Respondents indicating “sometimes, frequently or always.”

Interactions ranged from unsolicited “hallway” interactions to scheduled meetings [29],[30]. Representatives reached multidisciplinary US prescribers (MDs, NPs, PAs, and PharmDs) in 2009 through e-mail, fax, direct mail, cold calling, and social visits [26]. Face-to-face meetings with representatives were described by a purposive sample of UK nurse prescribers in 2003 as a rich and convenient source of drug information; pharmaceutical representatives were the most common source of information used prior to prescribing and were the most common source from whom nurse prescribers first heard about products [31],[32]. For a convenience sample of US psychiatric Clinical Nurse Specialists in 2004, the most common industry interaction was also with drug representatives, who were generally welcome anytime because of the perceived need for sample medications [33]. The reported main purpose of visits with infant formula representatives for staff at a UK health center was the acquisition of product information, educational updates, and infant nutrition support [29].

#### Gifts

The majority of sampled nurses, NPs, and RNs reported receiving gifts, food, and/or beverages, including sponsored lunch and dinner events [25],[27],[34] (Table 3). Thirty-six percent (*n = *33/92) of a convenience sample of US nurse prescribers in 2009 reported that sales representatives suggested some form of compensation (food or gifts) in exchange for preferential prescription of their product [35]. Among a random sample of family NPs (*n = *84) in 2009, those who were less critical of the practice of receiving gifts from industry accepted gifts more frequently (*r* = −0.48, *p*<0.0001) [34]. At community health facilities in Glasgow in 2007, 7% (*n = *48/669) of clinicians reported receiving gifts such as calendars, toys, meals, coffee, and coupons from the infant formula industry [29], while clinicians at 16% (*n = *5/32) of facilities in Burkina Faso in 2003 had received similar personal and professional gifts branded with “Nestle” or “Danone” [36].

#### Samples

The receipt and distribution of samples of pharmaceuticals and other medical products destined for patient use was reportedly prevalent across disciplines. The one exception is infant formula samples in countries that have enacted legislation compliant with the World Health Organization's International Code of Marketing of Breast-Milk Substitutes [37]. For example, by 2003, only 9% (*n = *1/11) of health facilities in Togo (legislative measures awaiting approval) and 13% (*n = *4/32) of facilities in Burkina Faso (enacted law) had received infant formula samples in the past 6 mo [36]; by 2007, 0.1% (*n = *9/669) of clinicians in Glasgow (many provisions enacted as law) had been given formula samples or feeding equipment in the past 6 mo [29]. However, in the US, which has taken no action on the code, 86% (*n = *44/51) of facilities in Massachusetts, US, in 2007 distributed industry-sponsored diaper bags (frequently containing infant formula and other product samples such as bottles) to new mothers [38]. Several of these facilities also reported the receipt of sample packs from formula companies that had been specifically designed for nurses [38].

Drug samples were prevalent in clinical practice. The proportion of clinicians reporting the acceptance and/or use of samples ranged from 66% to 86% (Table 3). Samples were dispensed by physicians, PAs, NPs, RNs, medical assistants, and office staff in 20% (*n = *314/1,588) of observed patient encounters in US primary care practices in 2000 [30]. Sample closets in these practices commonly contained very limited selections of medicines, largely representing only new, brand-name products. In less than half of observed patient encounters where samples were dispensed were samples accompanied by specific instructions, with dosing as the main focus [30]. None of the studied practices tracked serial numbers in the event of a medication recall; personal use of samples by clinicians was documented in 22% (*n = *4/18) of practices [30]. In 2011, US primary care prescribers (MDs, NPs, and PAs) working in for-profit clinics were significantly more likely to have samples available than those working in non-profit clinics (94% versus 50%, *p*<0.01) [39].

#### Education

“Educational” interactions with industry may be one of the most common ways that non-physician clinicians interact with industry, with proportions of clinicians reporting attendance at educational events or receipt of educational materials as high as 96% and 100%, respectively (Table 3). Industry filled notable resource gaps, providing funding to attend educational events and “information” suitable to a non-physician scope of practice and specific patient populations. For example, in 2003, pharmaceutical representatives supplied nurse prescribers in the UK with convenient pocket-sized cards containing all the information necessary to complete a prescription [32]. Historically, in 1966, a convenience sample of 1,080 hospital pharmacists received on average 24 pieces of direct mail advertisements a week [28].

A substantial proportion of industry-provided information is targeted at patients, but distributed, like product samples, through clinicians. In a convenience sample of senior RNs in New Zealand in 2009, 100% (*n = *120/120) of respondents had contact with drug industry information including package inserts, drug information sheets, sponsored patient education materials, article reprints, or drug company websites [27]. Among multidisciplinary staff at community health facilities in Glasgow in 2007, only a small minority had received funding to attend a conference, but 21% (*n = *137/669) had received industry literature on products, breast-feeding, becoming a dad, toilet training, and behavior management, and 1/3 of facilities had materials visible in patient care areas that were not compliant with the World Health Organization International Code of Marketing of Breast-Milk Substitutes [29]. Similarly, in West Africa in 2003, “educational” materials were found in 16% (*n = *7/43) of health facilities, none of which were code compliant, as they failed to mention factual information about the negative effects on breast-feeding when breast-milk substitutes are introduced, or the health hazards of breast-milk substitutes [36].

### Attitudes toward Industry

Nine studies explored clinicians' self-reported attitudes toward industry and interactions with industry. Although clinicians expressed a range of attitudes toward industry interactions, only a minority held negative views of industry; most clinicians across disciplines held favorable views of interactions with sales representatives and of industry interactions in general. The majority of a convenience sample of US nurse prescribers in 2009 described sales representatives as friendly and sociable (97%; *n = *89/92), professional (89.1%; *n = *82/92), and knowledgeable about their product (88%; *n = *81/92) [35]. About half (*n = *869/1,640) of a nationally representative sample of US pharmacists in 2005 endorsed an overall positive perception of the pharmaceutical industry, and half (*n = *853/1,640) disagreed with the statement that representatives were of little value to pharmacists [40]. US prescribers (MDs, NPs, PAs, and PharmDs) in 2009 enjoyed the easy and timely access to information about new and old drugs, and particularly enjoyed the samples, supplies, and food, that came with representative visits [26]. Among the specific benefits of interactions discussed, some prescribers emphasized the value of the social aspect of these interactions [26]. However, attitudes toward interactions with sales representatives appeared less favorable when they occurred more frequently; only 50% (*n = *46/92) of a convenience sample of US nurse prescribers in 2009 reported that sales representatives were considerate of their time [35]. Perceived resource gaps filled by industry included the provision of information suitable to the practice setting and to the scope of nurse prescribers [32] and drug affordability offset by samples [33]. Factors associated with more positive views toward industry included practice setting: pharmacists in university settings held more favorable attitudes toward industry, while those in managed care held the least favorable attitudes [40].

Other clinicians, however, characterized industry as an “important evil” [41] and were skeptical of the “marketing bent” of industry information [33]. The majority of clinicians in two US studies surveying pharmacists (2005) [40] and nurse prescribers (2009) [41] identified issues underlying negative or ambivalent attitudes toward industry, including excessive marketing [40], excessive drug prices [40],[41], provision of gifts that have nothing to do with patient care [40], and lack of industry information on marginalized populations such as geriatrics [41]. In one study in 2009, non-physician prescribers were offended when representatives did not interact with them, perceiving themselves to be treated as inferior to physicians [26]. Nevertheless, only 15% (*n = *14/92) of a convenience sample of US nurse prescribers in 2009 wished to discontinue their visits with sales representatives [35].

#### Attitudes toward samples

Clinicians had favorable views of free product samples. Nurse prescribers in the UK in 2003 greatly appreciated product samples such as wound care products, as they were able to become familiar with the product and to handle it themselves [32]. Seventy-three percent (*n = *192/263) of a random sample of US NPs in 2010 felt that medication samples were somewhat or very helpful in learning about new drugs [25]. Multidisciplinary US prescribers (MDs, NPs, PAs, and PharmDs) in 2009 enjoyed the convenience of samples and frequently referred to work with indigent or underinsured populations in citing their benefits [26]. These same prescribers, as well as a convenience sample of US gerontological NPs, however, acknowledged the difficulties in starting a patient on a brand-name drug they later would not be able to afford [26],[41]. The availability of samples in a clinic was associated with attitudes toward samples: prescribers with samples available were more likely to hold positive views of samples (*p*<0.01) [39]. For example, in Vermont, US, in 2011, primary care prescribers (MDs, NPs, and PAs) (*n = *206) who had samples available were significantly more likely to believe that patients liked samples and that samples helped patients who could not afford medication, reduced patient costs, and helped clinicians assess medication efficacy (*p<*0.01); prescribers without samples available were significantly more likely to believe that samples were overused, altered treatment plans, and increased costs of care (*p<*0.01) [39]. These attitudes appear to have changed little over time—in 1966, 85% (*n = *411/484) of a convenience sample of US hospital pharmacists considered drug samples to be valuable [28].

### Ethical Acceptability

Three studies assessed clinicians' perceptions of the ethical acceptability of industry interactions. Perceived ethicality of industry interactions differs from attitudes toward these interactions, as researchers have found that although clinicians may view industry interactions favorably, they may also judge them as unethical; nevertheless, they continue to engage in these interactions [26]. Overall, prescribing nurses felt the receipt of industry gifts to be more acceptable than did non-prescribing RNs; however, both groups of nurses felt it ethical and acceptable to attend sponsored events, particularly if they were “educational.”

The majority of two random samples of US NPs (total *n = *347) believed that the practice of gift-giving to NPs by sales representatives was ethical and acceptable [25],[34]. US family NPs in 2009 viewed gifts that were educational, were inexpensive, or had patient benefit as more ethical and appropriate than those without these attributes. These NPs afforded a high degree of acceptability to sponsored conferences or speakers [34]. However, many clinicians were also undecided or conflicted as to the ethicality of receiving gifts. Of a random sample of family NPs, 21%–33% (*n = *18–28/84) responded “no opinion” to statements about the ethicality of receiving various gifts [34]. Only a minority (35%; *n = *42/120) of a convenience sample of senior RNs in 2009 felt it acceptable for RNs to receive gifts, and nearly half (*n = *47/101) believed there should be conditions placed on the receipt of gifts, such as a value limit [27].

The majority of nurses sampled believed it appropriate to attend sponsored meals and educational events, or to accept funding to do so [25],[27],[34]. Though likely of greater cost, a representative sample of family NPs in 2009 rated sponsored lunches and dinners as more appropriate than “happy hour” events [34]. The majority of a sample of senior RNs in New Zealand in 2009 (70%; *n = *84/120), though less comfortable with receiving gifts, felt it acceptable to receive drug company funding to organize or attend a conference [27]. RNs' open-ended comments justified these views, suggesting that industry funding was the only remedy for significant resource gaps; there was also the prevailing view that because physicians attended sponsored events, received funds, and accepted gifts, it was appropriate for nurses to do so as well [27].

### Perceived Impact of Industry Interactions on Clinician Practice

Six studies assessed clinicians' perceptions of the impact of industry interactions on their or their colleagues' practices. These perceptions were measured using self-reported practice behaviors or responses to hypothetical clinical scenarios. Only a minority of clinicians perceived that industry marketing influenced their own practice, even when acknowledging studies to the contrary [26],[34]. However, two-thirds (*n = *71/106) of a convenience sample of New Zealand RNs in 2009 suggested a positive impact on their practices, stating that drug industry information probably or definitely improved their practice because of its educational value [27]. While denying that marketing could influence their own practices, a significantly larger percentage of clinicians felt their colleagues would be influenced [34]. For example, only 6% (*n = *5/84) of a systematic random sample of US family NPs in 2009 perceived themselves to be influenced by pharmaceutical representatives, but 21% (*n = *18/84) believed other advanced practice nurses to be influenced, and 24% (*n = *20/84) believed physicians were influenced more than NPs [34]. A nationally representative sample of US NPs in 2010 (*n = *263) was more specific about the perception of influence of marketing based upon the type of marketing: though 93% (*n = *245/263) reported that free gifts had no effect on their likelihood to prescribe a highlighted drug, about 2/3 (*n = *181/263) felt that sponsored meals encouraged prescribing of new, highly marketed drugs, with just under half (*n = *126/263) reporting they were personally more likely to prescribe a highlighted drug after attending a sponsored event [25]. NPs were more willing to agree that drug samples affected their prescribing practices; 62% (*n = *163/263) of randomly sampled US NPs in 2010 indicated that samples influenced their choice of medication [25]. In 1966, 40% (*n = *194/484) of a convenience sample of US hospital pharmacists indicated that receipt of drug samples influenced the acceptance of a drug into the hospital's formulary [28].

In 2011, primary care prescribers (MDs, NPs, and PAs) working in Vermont, US, were asked to self-report prescribing practices in response to hypothetical vignettes. Clinicians without samples available in their clinics were significantly more likely to report they would prescribe the medication recommended by the evidence-based clinical guidelines or the generic alternative in response to the hypothetical scenarios for hypertension and depression [39]. Controlling for for-profit setting, practice size, and demographics, clinicians with samples available were significantly less likely to report willingness to prescribe the guideline-recommended thiazide diuretic for treatment of hypertension (odds ratio [OR] = 0.15, 95% CI 0.04–0.56); those who dispensed samples at least weekly were less likely to select the guideline-recommended thiazide diuretic than those that dispensed samples less frequently or not at all (OR = 0.4, 95% CI 0.18–0.85) [39].

### Perceived Reliability of Industry Information

Eight studies assessed clinicians' perceptions of information provided by industry, including the perception of bias, objectivity, and comprehensiveness. Though a sizable proportion of clinicians across disciplines questioned the reliability of industry information, the majority remained confident that they could detect biased information and thus rated this information as reliable, valuable, or useful [25]–[27],[31],[32],[40]. Only 18% (*n = *18/103) of a convenience sample of New Zealand RNs in 2009 felt they had probably or definitely been exposed to misleading information; however, 50% (*n = *51/103) felt they probably or definitely would recognize biased information if present [27]. These RNs were significantly more confident in their own ability to detect bias than in their peers' ability to do so (OR = 3.19, 95% CI 1.95 to 5.20) [27]. A purposive sample of UK nurse prescribers in 2003 used pharmaceutical representatives as one of the most common sources for drug information, followed by colleagues; although recognizing the potential for bias, both they and their managers were confident in their ability to detect biased information and cited strategies such as requesting evidence for benefit prior to prescribing [31],[32]. Multidisciplinary clinicians at Glasgow health centers in 2007 felt that formula industry information was essential in providing care to bottle-feeding mothers and that this information was needed to keep clinicians up-to-date [29].

In contrast, although most of a convenience sample of US nurse prescribers in 2009 believed that industry representatives were knowledgeable about the medication they promoted, nearly 30% (*n = *26/92) reported that these representatives could not answer questions about their product [35]. Other issues that nurse prescribers raised included representatives' difficulties in discussing the positive and negative aspects of medications equally well, and representatives' lack of understanding as to the dangers associated with mixing incompatible medications [35].

Several factors may be related to clinicians' perception of bias. Over 2/3 (*n = *1,132/1,640) of a random sample of US pharmacists in 2005 felt that information provided by industry would have greater value if the sales representative was a health care professional [40]. Of interest, a convenience sample of hospital pharmacists in 1966 (*n = *1,080) expressed similar views. Although endorsing “detailmen” as the best source of drug information, many were critical of the “sales pitch” and would prefer to see companies hire pharmacists in this role [28]. Similarly, the provision of continuing education credits increased NPs' perceptions of the reliability of information provided at sponsored lunch or dinner events [25]. Belief in the value of industry information was positively correlated with extent of industry involvement for a convenience sample of senior RNs in New Zealand [27]. For example, RNs who had attended sponsored events were substantially more likely to believe in the value of industry-derived information (OR = 4.81, 95% CI 1.60–14.46) [27].

### Preparation for Industry Interactions

Four studies assessed whether clinicians had been prepared for how to interact with industry in their professional training [33],[34],[36],[40]. Overall, preparation for industry interactions was not a part of professional training. A systematic random sample of US family NPs in 2009 was evenly divided between those who felt they had been educated about industry interactions and those that did not. There was no correlation between years of experience and attitudes toward industry or industry involvement [34]. In the US in 2004, a small convenience sample of nurse prescribers reported having had no preparation as to how they should interact with industry in their clinical practice [33]. Where professional or regulatory guidelines for interactions existed, clinicians lacked awareness and training. In West Africa in 2003, nearly 80% of providers at community health facilities had never heard of the World Health Organization International Code of Marketing of Breast-Milk Substitutes; of those who had, only 63% had read any or all of it, and only 2% in either Burkina Faso or Togo had received any training on it [36]. Practice setting may be associated with knowledge of guidelines: among a random sample of US pharmacists, those working in university settings were significantly more knowledgeable about the Code on Interactions with Health Care Professionals created by the trade association Pharmaceutical Research and Manufacturers of America than those in managed care or community settings (*p*<0.001) [40].

### Reaction to Industry Relations Policy

Three studies assessed clinicians' reactions to policies designed to guide industry–clinician interactions. These policies were largely institutional. Generally, clinicians were not in favor of the implementation of policy guiding industry interactions and resented the limitations placed upon these activities. A small convenience sample of nurse prescribers in the UK in 2003 who were subject to a ban on individual meetings with representatives felt they were missing a valuable source of information [31]. Some community health clinic staff in Glasgow in 2007 expressed concern that policy promoting breast-feeding had “gone too far” and that dangerous bottle-feeding practices might result because of lack of targeted information such as that provided by the formula industry [29]. Among a multidisciplinary focus group of prescribers (MDs, NPs, PAs, and PharmDs) in 2009, very few had participated in developing policy for industry interactions, and most acknowledged simply adjusting to the norms of their practice setting; where policies aimed to limit interaction, prescribers acknowledged that representatives still made it very easy to meet [26]. Though some believed existing limitations on industry interactions were unfair, others welcomed limitations, realizing that over time their prescribing had been influenced by sales visits and sample availability [26]. A unique finding was that policies were often implemented differentially across disciplines (e.g., a policy that restricted meetings with prescribers, but where representatives continued to meet with RNs) [26].

### Managing Industry Interactions

Three studies assessed how clinicians managed industry interactions. Although formal strategies were reported in two studies, the majority of clinicians or practice settings studied did not adopt these, and often these strategies were perceived to be inadequate in minimizing patient care disruptions. For example, clinicians at about half the US primary care practices purposively surveyed in a 2000 study had formal strategies for guiding interactions with drug representatives [30]. These strategies were used to minimize disruption to clinic activities or distraction of clinicians, and included scheduling specific times for industry interactions, making clinicians' schedules available to representatives, and scheduling representatives as if they were patient appointments; however, at most practices, interactions reportedly remained haphazard and counterproductive to both clinicians (e.g., interruption of patient care activities) and representatives (e.g., lack of clinician attendance at events) [30]. Occasionally, formal strategies developed for meetings with representatives failed: 36% (*n = *33/92) of a convenience sample of US nurse prescribers in 2009 reported that representatives failed to observe the rules set for the visit [35].

However, for most surveyed clinicians, strategies for managing interactions with industry were informal and poorly articulated, determined by the norms of the practice setting [26],[30]. Multidisciplinary US prescribers (MDs, NPs, PAs, and PharmDs) during focus groups in 2009 justified their meetings with sales representatives, despite feeling discomfort with the actual interactions, by claiming to be skeptical [26]. Key reasons for continued interaction were a social climate that dictated they not be “rude,” having long-term relationships with representatives, and not wanting to disrupt practice norms [26]. Prescribers, however, remained largely confident that they could manage these interactions effectively without introducing bias or conflict of interest into practice [26].

## Discussion

While non-physician clinicians have been largely omitted from research and policy on industry interactions, it appears that nurse prescribers including NPs, and perhaps RNs, pharmacists, midwives, and dieticians, interact frequently with industry in numerous ways. There is evidence to suggest that not only the pharmaceutical industry, but also the infant formula industry target marketing efforts at non-physician clinicians. The frequency of industry interactions and, despite clinician recognition of the potential for bias and conflict of interest, the common view of industry as, at worst, a “necessary evil” suggest that clinician–industry interactions are normalized in clinical practice settings.

Non-physician clinicians are positioned much differently within the health care system than their physician counterparts, both in the way their employment is structured and in their relationship to patients. For example, RNs are situated at important junctions of decision-making in the delivery of patient care, as they are responsible for coordinating, planning, and evaluating care in collaboration with multidisciplinary teams, patients, and families. This suggests that nurses, pharmacists, and other allied health professionals may be well positioned to act as vehicles for industry marketing to both prescribers and to patients. In order to maximize profits for shareholders, pharmaceutical companies must not only maximize the number of new prescriptions, but also ensure adherence to prescribed medications and shorten the time between identifying a condition and getting a prescription to treat it [42]. Nurses and pharmacists are key actors in the promotion of medication adherence and the coordination of care; the body of existing research reviewed suggests a “marketization” of this nursing activity by the pharmaceutical industry as companies attempt to “partner” with nurses to achieve what they portray as a mutual goal [9]. Increasingly, pharmaceutical companies are establishing compliance departments and engaging consultants to address losses incurred through patient noncompliance; nurses, pharmacists, and others acting as “gatekeepers” in the distribution channel for medications are brought into alignment with pharmaceutical industry goals through partnerships in the form of patient compliance programs [9],[43]. Further, mid-level prescribers such as NPs and PAs are increasingly seen as key prescribers by industry, as they are situated at the front lines of primary care and are open to pharmaceutical industry involvement in their practice [42]. Though this review has helped to establish that industry interactions among non-physician clinicians are common, further work is needed to understand how or if these interactions affect the cost, quality, and safety of patient care. Particularly needed are study designs that examine associations, longitudinal trends, and causation between non-physician clinician–industry interactions and purchasing decisions, prescribing, patient safety, and the quality of clinician and patient education.

Clinicians noted disparities between themselves and their physician counterparts in their access to practice resources, ability to afford to attend conferences, and perception that it was permissible to engage in marketing. In some ways, these perceived disparities made non-physician clinicians more amenable to interacting with industry, as they perceived industry interactions as a means to elevate their status within the health care system or to access similar perquisites and opportunities afforded to physicians (e.g., “but doctors do it” [27]). In order to address some of these perceived disparities, policies such as the Physician Payments Sunshine Act could be made inclusive of all clinicians.

Thus far, relative to the pharmaceutical industry, researchers and policymakers have overlooked the activities of the medical device industry. Given the high cost and nonrational prescribing resulting from physician–pharmaceutical industry interactions [44]–[46], a plausible hypothesis is that similar industry interactions between clinicians and the device industry may similarly affect purchasing decisions. The paucity of research on the activities of the device industry is a notable gap in the literature and one that may have significant implications for the cost, quality, and safety of care. For example, the increasing costs associated with implants used in hip and knee surgeries prompted action on the part of the US Department of Justice resulting in civil settlements, compliance regulations, and federal oversight of payments made by device manufacturers to orthopedic surgeons [47]. Further research is required to explore whether and how the medical device industry markets its products to clinicians and to understand the impact of these activities on clinicians' purchasing decisions. Although unstudied to date, the role of nurses in purchasing decisions (e.g., operating room nurses are frequently in charge of these budgets) may be one of the most important ways in which nurses interact with industry. As “end users” of many medical devices and supplies ranging from wound care products to high-tech hemodynamic and cardiac monitoring systems, nurses are frequently exposed to industry representatives to maintain competence with existing and new equipment. This type of interaction has been omitted from research and policy addressing industry–clinician interactions, and thus whether and how to monitor and guide such interactions remains unclear. Further, given the aging population, many other industries that frequently interact with non-physician clinicians, including the home health and long-term care industries, may also come under increased scrutiny for their roles in issues around cost, quality, and safety, and research into these interactions would be beneficial. No studies of clinician interactions with these other industries were identified by this review.

### Limitations

Due to the variation in study design, the lack of standardized measurement tools to assess outcomes, and the inclusion of qualitative reports, quantitative analysis of the data from retrieved studies was infeasible. Because of the observational and qualitative nature of the studies reviewed, the prevalence or frequency of non-physician clinician–industry interactions could not be quantified. Because of the absence of experimental, cohort, or case control studies and the variety of designs employed, established scales used to assess methodological quality for inclusion in systematic reviews were not appropriate. Thus, the findings derived from this systematic review are based on studies with varying methodological rigor. However, systematic reviews employing qualitative synthesis rarely exclude studies based on methodological quality scores [48],[49]. This review is the first to our knowledge to provide a descriptive analysis of the literature on non-physician clinician–industry interactions.

None of the studies found in the search examined the association between industry interactions and patient care outcomes. The only studies identified in the search that examined the association of industry interactions with patient care had physician-only samples and thus were excluded on this basis. Although these studies may be replicated with non-physician prescribers, the challenge will be to identify measurable outcomes to relate industry influence on non-prescribing clinicians to the cost, quality, and safety of care.

### Conclusions

While some aspects of clinician–industry interactions may be beneficial, the normalization of such relationships in clinical settings creates the potential for serious risks for patients and health care systems. Yet it may be unrealistic to expect that clinicians can be taught individually how to interact with industry ethically or to detect and avert bias. Social science researchers suggest that the rational choice view of conflict of interest does not reflect the evidence, arguing that judgments are subject to a “self-serving” bias that is both unconscious and unintentional [50]. The problem of self-serving bias suggests that clinician education will not be effective in mitigating unconscious biases, nor will disclosure be an effective means to counteract biases [50]. Further, even clinicians who consciously seek to avoid interactions with industry may fail because of the ubiquitous nature of marketing and promotional materials [51] and the strength of practice and social norms [26]. Although education alone may be ineffective, the ethical implications of such interactions could be problematized for clinicians during professional and continuing education, and issues such as the introduction of bias into clinical decision-making could be addressed at an institutional or regulatory level. Policy recommendations include extending the Physician Payments Sunshine Act to include all clinicians and ensuring that institutional industry relations policies are inclusive of all disciplines. Rather than relying on the judgment of individual clinicians, the environment in which clinicians practice should be structured to mitigate the potentially harmful effects of industry involvement in health care.

## Supporting Information

Text S1 **PRISMA checklist for content of a systematic review.**
(PDF)

## Abbreviations

MD: Doctor of Medicine
MeSH: Medical Subject Headings
NP: Nurse Practitioner
OR: odds ratio
PA: Physician Assistant
PharmD: Doctor of Pharmacy
RN: Registered Nurse
